# Quenching and Tempering-Dependent Evolution on the Microstructure and Mechanical Performance Based on a Laser Additively Manufactured 12CrNi2 Alloy Steel

**DOI:** 10.3390/ma16093443

**Published:** 2023-04-28

**Authors:** Wei Zhang, Xin Shang, Xiaoxuan Chen, Shenggui Chen, Zhengliang Liu, Lijuan Zhang

**Affiliations:** 1 Institute of Science & Technology Innovation, Dongguan University of Technology, Dongguan 523820, China; zw18640457640@163.com (W.Z.); shangxin0375@126.com (X.S.); celiex@163.com (X.C.);; 2 School of Materials Science and Engineering, Tianjin University, Tianjin 300350, China; 3 School of Art and Design, Guangzhou Panyu Polytechnic, Guangzhou 511483, China; 4 Songshan Lake Materials Laboratory, Dongguan 523808, China; iuzhengliang@sslab.org.cn

**Keywords:** directed energy deposition, tempering, microstructures, mechanical property, low-carbon low-alloy steel

## Abstract

For exploring an effective heat treatment schedule to enhance the strength–plasticity balance of the ferrite–austenite 12CrNi2 alloy steel additively manufactured by directed energy deposition (DED), 12CrNi2 was heat-treated with deliberately designed direct quenching (DQ) and cyclic quenching (CQ), respectively, and the differently quenched steels were then tempered at a temperature from 200 °C to 500 °C. It was found that the CQ, in contrast to the DQ, led the 12CrNi2 to have significantly increased tensile strength without losing its plasticity, based on the introduction of fine-grained lath martensite and the {112}<111>-type nanotwins. The nanotwins were completely degenerated after the 200 °C tempering. This led the CQ-treated steel to decrease in not only its tensile strength, but also its plasticity. In addition, an interesting phenomenon observed was that the DQ-induced laths and rod-like precipitates, and the tempering-induced laths and rod-like precipitates were all prone to be generated along the {112} planes of the martensitic crystal (α-Fe), which were exactly fitted with the {112}-type crystalline orientation of the long or short nanotwins in the CQ-induced martensite. The quenching–tempering-induced generation of the {112}-orientated laths and rod-like precipitates was explicated in connection with the {112}<111>-type long or short nanotwins in the CQ-induced lath martensite.

## 1. Introduction

Ever-increasing demands for high-precision shape control and high-freedom structural design during the manufacturing process of the structural components led the conventional metallurgical fabrication technologies (e.g., forging, casting, rolling and extrusion) to hardly satisfy the design requirements for forming structural parts with a large size and complex shape. Additive manufacturing (AM) has been more and more popularly introduced for the manufacture of complex components in a point-by-point, line-by-line and layer-by-layer manner within a short time [1]. This established new field for manufacturing the structural parts, which were difficult or impossible to manufacture using the conventional metallurgical techniques, made the structural design not limited by processing technologies [2,3]. AM could be divided mainly into powder bed fusion (PBF) and directed energy deposition (DED) processes for metal alloys. The DED process is not limited by the size of the forming cavity, and hence it is suitable to form large-sized structural components.

Many typical alloys, such as stainless steels, alloy steels, titanium alloys and superalloys are fabricated via DED into a variety of components with different requirements. Among them, 12CrNi2 is a low-carbon low-alloy steel feature with low cost, good processability and an acceptable indicator of mechanical property. It has been validated in typical applications of structural parts, such as diesel engine shafts, which are large in size and are complex constructions. Recently, the utilization of the DED to fabricate 12CrNi2 has been investigated by some researchers [4,5,6]. The as-deposited 12CrNi2 was characterized as having a microstructure consisting of dual phases of major ferrite and minor austenite [7]. This led the 12CrNi2 to have excellent ductility but poor strength, which could hardly withstand the severe loading produced in the service condition. However, at present, considerable progress has not been achieved in the DED-manufactured low-carbon low-alloy steels in the acquisition of not only expected ductility, but also high strength, due to the difficulty in controlling the thermal cycle history and the complicated liquid–solid and solid–solid transformations during the rapid layer-by-layer deposition of the DED [8]. Under this background, the post-heat treatment is hereby an important way to optimize the microstructure, so as to achieve desirable mechanical properties of the additively manufactured 12CrNi2.

It is widely acknowledged that quenching leads the steel to generate a hard phase, i.e., lath martensite, which helps to improve the strength of the as-deposited 12CrNi2. However, a poor plasticity concomitantly occurs. This is deemed to originate from its massive multi-scale interfaces within the lath martensite [9,10]. The lath martensite in the low-carbon as-quenched steel was always separated by prior austenitic grain boundaries. The prior austenitic grain consists of packets sharing the same habit plane of γ-Fe. Each packet is divided into blocks, and each block is further subdivided into a group of laths having a small misorientation between one other. Tempering followed quenching at lower temperatures is always adopted to toughen the as-quenched steel, and hence it provides a flexible combination of strength and ductility. The mechanical performance of the as-tempered martensite intrinsically attributes the evolution of the as-quenched lath martensite during tempering. The lath martensite exhibited high-density boundaries which had a high-interface energy naturally providing a driving force for the boundary arrangement and grain growth during the tempering, and hence weakening the grain boundary-strengthening effect on the steel [11]. Moreover, the substructure inside the martensitic laths were always abundant with line defects, such as dislocations, which were naturally produced by the rapid cooling during the water quenching. The high-density defects exhibited a strong tendency in the dislocation recovery and grain recrystallization, which could annihilate some of the sites for carbon atom segregation, and hence promote the local precipitation of various types of carbides [12]. As such, the above tempering-induced structural evolutions led the steel to have enhanced ability of the continuous plastic deformation through the dislocation motion which was always easier, because the dislocations have been released during the tempering [13]. Concurrently, the tensile strength was inevitably decreased. In brief, the strength–plasticity trade-off of the heat-treated steel was always difficult to break through. 

In previous decades, many typical heterogeneous structures, such as bimodal grains, nano-precipitate and gradient nano-structure [14,15,16], were artificially introduced to break this inversion relationship between strength and plasticity. In addition, the body-centered cubic (BCC) {112}<111>-type nanotwins were observed in the martensitic laths of the as-quenched Fe–C-based alloys having low carbon and low alloy content, such as Fe-0.05C (wt.%), Fe-0.1C (wt.%) and Fe-Ni-0.08C (wt.%) in recent years, [17,18,19]. It has been noticed that the nanotwins could provide a strong strengthening effect through retarding the dislocation motion [20]. Concurrently, the nanotwins having smaller twinning shear and lower magnitude of shape strain were also expected to be beneficial structures for the plastic deformation. Additionally, some other reports also declared that the nanotwins in the lath martensite were favorable to disperse the local concentrated strain, because the nano-twinned boundaries could be used as effective paths for dislocation gliding, and hence accommodated more strain there [21,22]. Thus, the introduction of nanotwins in the lath martensite could be an effective strategy to enhance the strength–plasticity balance of the low-carbon low-alloy steel. As stated previously, the nanotwins have been acquired in the low-carbon and low-alloy martensitic steel by some researchers. However, no thorough investigations were conducted into the twinning mechanism during quenching, and the evolution of nanotwins during the following tempering in terms of the {112}<111>-type nano-twinned lath martensite in the low-carbon and low-alloy Fe-C based alloys. 

Based on the analysis above, high-density {112}<111>-type nanotwins were introduced into the lath martensitic microstructure of the DED additively manufactured 12CrNi2 through a deliberately designed cyclic quenching (CQ) treatment. To gain further insight into the evolution of the nanotwins inside the lath martensite during the tempering, tempering at various temperatures was then conducted on the CQ-treated 12CrNi2. For comparison, the 12CrNi2 having the dislocation-dominated lath martensitic microstructure was going to be produced through a direct quenching (DQ). The DQ-treated 12CrNi2 was also tempered at the same temperatures as those applied to the CQ-treated one. The quenching–tempering-dependent evolution mechanism of the nanotwins in the martensitic laths was analyzed in connection with the morphology and crystallography characteristics of the as-tempered martensite. The microstructure-dependent mechanical performances of the heat-treated 12CrNi2 were then discussed in detail. The results could offer a new guidance for controlling the heterogeneous substructures of the lath martensite through the explored quenching schedules, and hence improving the strength–plasticity balance of the laser additively manufactured 12CrNi2. This has not been reported by the established literatures. This work also has importance for widely used applications when considering the developments of DED on the low-carbon low-alloy steel structural parts.

## 2. Materials and Methods

**Figure 1:**
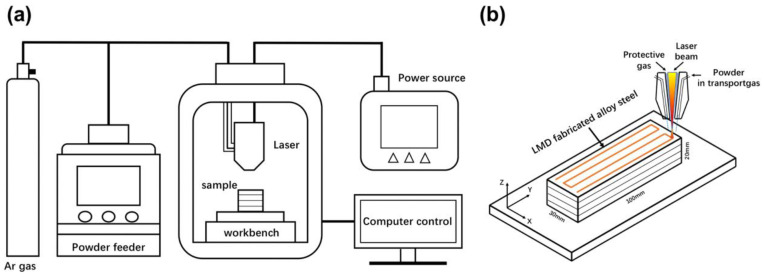
The schematic diagrams of (**a**) DED system and (**b**) DED process.

The low-carbon low-alloy 12CrNi2 steel powder was commercially produced by gas atomization. The chemical composition of the powder was 0.14–1.72Ni-0.82Cr-0.18Si-0.54 Mn-0.021O balanced with Fe in mass percent. The powder used was with an average particle diameter of about 150 μm. The cuboid-shaped 12CrNi2 alloy steel with dimensions of 100 × 30 × 20 mm^3^ was laser additively manufactured by DED on a Laser Melting Deposition 8060 system equipped with a Laserline LDF 3000-60 semiconductor laser device. The schematic diagrams of the DED system and the DED process are shown in Figure 1. The laser beam, as a high-energy heat source, cyclically scanned along X and Y directions on the two-dimensional plane parallel to the substrate, and acted on the powders which were synchronously transported by the powder-feeding device. The substrate material was the commonly used Q235 steel. Concurrently, the molten pool was deposited layer-by-layer along the Z direction as dictated by a computer-aided design (CAD) 3D file. The Z direction was perpendicular to the X–Y two-dimensional plane as displayed in Figure 1b. The laser power was 900 W, the laser scanning speed was 600 mm/min, the laser beam spot diameter was 2 mm, the powder feeding rate was 7 g/min, the layer thickness was 0.65 mm, the hatch distance was 1 mm and the overlap rate was 50%. The DED process was performed in the carrier gas of high purity Ar atmosphere (O_2_ level < 20 ppm). To conduct the heat treatments and the microstructure inspections, small cuboid-shaped specimens of 5 × 5 × 8 mm^3^ were then cut from the as-deposited 12CrNi2 block. 

**Figure 2:**
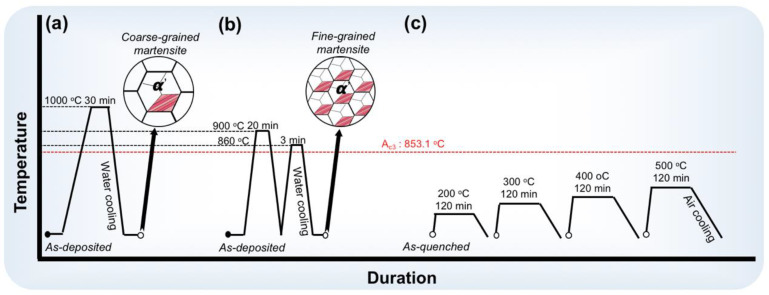
Detailed schematic diagrams of the heat treatments including (**a**) direct quenching (DQ), (**b**) cyclic quenching (CQ) and (**c**) tempering.

To introduce the lath martensite having various substructures of nanotwins and dislocations, respectively, the small specimens were subjected to two deliberately designed quenching treatments at the temperatures above A_c3_, as schematically illustrated in Figure 2. The A_c3_ (853.1 °C) of the as-deposited 12CrNi2 was measured by a L78 RITA thermal expansion instrument. Some of the small specimens were heated to 1000 °C, and isothermally held for 30 min using the muffle furnace, and then quenched into water as illustrated in Figure 2a. The DQ was conducted on the steel specimens, so as to obtain the coarse-grained lath martensite having a dislocated substructure. In contrast, the CQ was deliberately designed on a basis of a lot of microstructural inspections of the as-quenched 12CrNi2 steel specimens to produce finer-grained lath martensite having numerous BCC {112}<111>-type nanotwins. The duration of the CQ treatment is displayed in Figure 2b. The small specimens were heated to 900 °C, and isothermally held for 20 min before quenching in the water. The as-quenched specimen was cyclically quenched after the austenitization at 860 °C for 3 min. Subsequently, the DQ-treated and CQ-treated small specimens were subjected to tempering at 200, 300, 400 and 500 °C for 2 h as described in Figure 2c.

**Figure 3:**
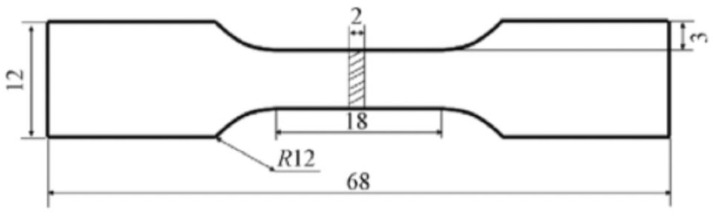
The shape and size of the tensile specimen. (All dimensions are in mm.).

To make the prior austenitic grain boundaries of the lath martensite clearly visible under an optical micrograph (OM), the differently quenched specimens were etched by saturated picric acid solution at a water bath temperature of around 65 °C for 1 min. The electron back-scattered diffraction (EBSD) examination was conducted on the two as-quenched specimens using TESCAN MAIA3XMH at an accelerating voltage of 20 kV, and a step size of 0.2 µm. The specimens for EBSD tests were prepared through fast mechanical grinding and the following polishing using silica suspension to remove the surface stress. The scanning electron microscope (SEM) was conducted on the as-quenched and as-tempered specimens etched by 4% acid–alcohol solution. The mean sizes of the prior austenitic grains, packets and blocks were determined from the measurements covering 30 prior austenitic grains, 50 packets and 50 blocks, using the image analyze software (IAS). The internal substructures of the as-quenched and as-tempered martensitic laths were inspected with a transmission electron microscope (TEM). The specimens for TEM tests were electro-polished in a twin-jet electro-polisher with a chemical solution of 10% HClO_4_ and 90% ethanol. In addition, tensile specimens with the specific shape and size shown in Figure 3 were cut from the as-deposited 12CrNi2 steel block. Tensile tests were conducted on the tensile specimens without or with the quenching and tempering treatments using the Instron 5982 at room temperature according to the GB/T 228.1-2010 Standard [23]. Three tensile tests were conducted on each type of specimen, such as as-deposited specimen, as-quenched specimen and the as-tempered specimen, in order to ensure the credibility and repeatability of the mechanical properties. 

## 3. Results 

### 3.1. Morphology and Crystallography of the As-Quenched Martensite

**Figure 4:**
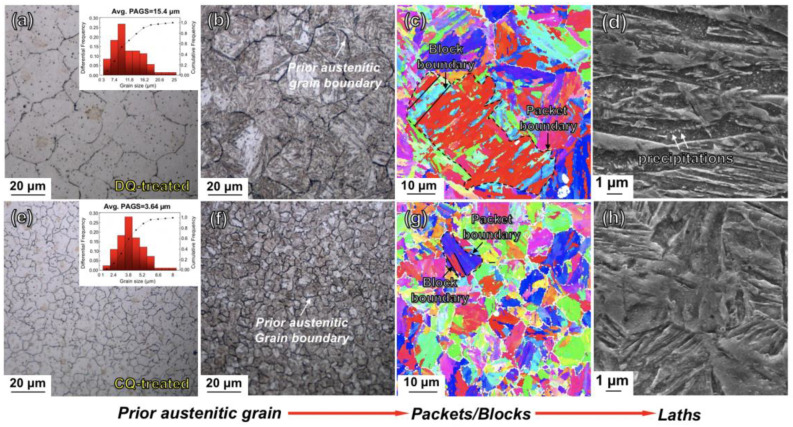
Hierarchical martensitic microstructures of DQ-treated and CQ-treated specimens: (**a**,**b**) and (**e**,**f**) optical micrographs revealing the prior austenitic grains of the DQ-induced and CQ-induced lath martensite, respectively; (**c**,**g**) EBSD inverse pole figures (IPFs) revealing the DQ-induced and CQ-induced martensitic packets and blocks, respectively; (**d**,**h**) scanning electron micrographs revealing the particles precipitated inside the DQ-induced and CQ-induced martensite laths, respectively.

The microstructures of the DQ-treated and CQ-treated specimens are displayed in Figure 4, which consists of typical optical micrographs, EBSD inverse pole figures (IPFs) and scanning electron micrographs. From the optical micrographs (Figure 4a,b,e,f), the two differently quenched martensitic microstructures were both characterized by the morphology of laths. The lath martensite exhibited the dark lines as the prior austenitic grain boundaries. The prior austenitic grains of the DQ-induced lath martensite were large-sized, and the mean size was measured to be ~15.4 μm using the IAS. In contrast, the CQ led the prior austenitic grains to be apparently refined (~3.64 μm). A prior austenitic grain was divided by several boundaries of polygonal-shaped packets (see the black dotted lines in the EBSD inverse pole figures (IPFs) in Figure 4c,g). From Figure 4c,g, each packet consisted of several blocks arranged parallel each other. The mean sizes of the prior austenitic grains, packets and blocks of the DQ-induced and CQ-induced lath martensite are listed in Table 1. It is apparent that the CQ also induced the decrease in the average sizes of both the packets and blocks. Moreover, one may note that many particles apparently precipitated inside the DQ-induced coarse-grained martensitic laths (see the typical scanning electron micrograph in Figure 4d). In contrast, few or no particles precipitated in the CQ-induced fine-grained martensitic laths as presented in Figure 4h. 

**Figure 5:**
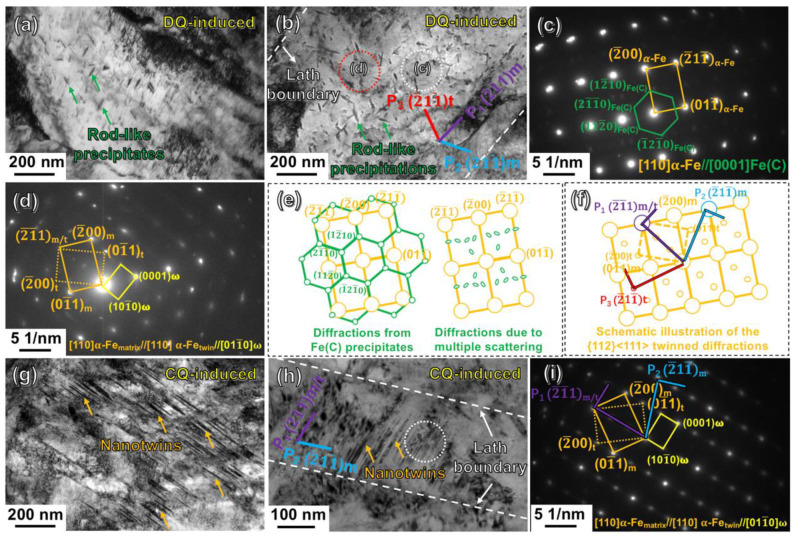
TEM observations of the DQ-treated and CQ-treated specimens: (**a**,**b**) bright-field images of the DQ-treated and CQ-treated specimens, respectively; (**c**,**d**) the corresponding selected area electron diffraction (SAED) patterns of the circled areas in (**b**); (**e**,**f**) the schematic diagrams of the SAED patterns in (**c**) and (**d**), respectively; (**g**,**h**) bright-field images revealing the nanotwins inside the CQ-induced fine-grained lath martensite; (**i**) the corresponding SAED pattern of the nano-twinned area in (**h**).

In order to further identify the substructures inside the martensitic laths, TEM determinations were carried on the DQ-treated and CQ-treated specimens. The TEM results were shown in Figure 5. It could be seen from the bright-field images (Figure 5a,b) that the particles precipitated inside the DQ-induced coarse-grained martensitic laths were dominantly rod-like in shape. The rod-like precipitates were further identified through the electron diffraction at the selected area circled by the white dotted line in Figure 5b, and the selected area electron diffraction (SAED) pattern was shown in Figure 5c. The major set of diffraction spots with higher brightness was indexed as the martensitic matrix of BCC structured α-Fe in [110] projection, and the extra spots with lower brightness originated from the Fe(C) precipitates. The Fe(C) was a hexagonal close-packed (HCP) phase with an orientation relationship with the martensitic matrix of α-Fe: [110]_α-Fe_ // [0001]_Fe(C)_, (001)_α-Fe_ // (1$\bar{2}$10)_Fe(C)_, on a basis of the study from Tirumalasetty et al. [24]. As schematically sketched in Figure 5e, the green spots connected by green lines were the primary diffractions (see the illustration at the left side), and the other smaller green spots were proved to be derived from the multiple scattering (see the illustration at the right side). Curiously, another diffraction pattern was also obtained through electron diffraction at another selected area circled by a red dotted line in Figure 5b. The pattern, as typically shown in Figure 5d, consisted of a set of major α-Fe_matrix_ spots, and a set of {112}<111>-type twinned α-Fe_twin_ spots. However, the quality of the α-Fe_twin_ spots was too poor, and the twinned morphology could be hardly distinguished in real space (Figure 5b), plausibly because the twin’s amounts were too few. Nevertheless, it could be deduced that the BCC structured {112}<111>-type twins have emerged in the coarse-grained martensitic laths during the DQ process. In addition, it could be clearly seen from Figure 5b that the rod-like Fe(C) precipitates dominantly generated along three planes of P_1_, P_2_ and P_3_ as marked by purple, blue and red lines in Figure 5b. The three planes were all indexed to be {112} planes of the α-Fe, on the basis of the relationship that the lattice plane in the real space was vertical to the connected line between the corresponding diffraction spot and the transmission spot in the reciprocal space. Accordingly, the P_1_ parallel to the lath boundary was determined as ($\bar{2}\bar{1}$1) of α-Fe_matrix_ (see the two purple colored vertical lines in the schematic illustration of Figure 5f), and the P_2_ aligned along the ($\bar{2}$1$\bar{1}$) plane of α-Fe_matrix_ (see the two blue vertical lines in Figure 5f). The P_3_ was measured to be suitably symmetrical to P_2_ with respect to P_1_. Referring to this correlation, the P_3_ was exactly parallel to the ($\bar{2}$1$\bar{1}$) of α-Fe_twin_ (see the two red vertical lines in Figure 5f). The α-Fe_twin_ is the twinned lattice of α-Fe_matrix_ related to the twinning boundary of P_1_, i.e., ($\bar{2}\bar{1}$1) (see the twinned relationship illustrated in Figure 5d).

Differently from the DQ-induced martensite, large amounts of nanotwins were frequently observed by TEM in the CQ-induced lath martensite. Figure 5g,h show the typical bright-field images of the long and short nano-twinned morphologies in the CQ-induced martensitic laths, respectively. Figure 5i displays the SAED pattern of the short nano-twinned area circled by white dotted line in Figure 5h. This pattern consisted of a set of spots of α-Fe_matrix_ and another set of spots of α-Fe_twin_ at the zone axis of [110]. The α-Fe_twin_ exhibited a definitely BCC-structured {112}<111>-type twinned relationship with α-Fe_matrix_. Additionally, another set of spots with lower brightness (see the spots connected by the yellow rectangular line in Figure 5i) was indexed to be an HCP-structured ω-Fe along the zone axis of [01$\bar{1}$0]. The ω-Fe has been firstly reported by Silcock et al. in the study of BCC-structured Ti, Zr-based alloys after rapid cooling or deformation since the 1950s [25]. These small ω-Fe particles in nano-scale size are always located at the boundaries of the BCC {112}<111>-type nanotwins [26,27]. The lattice parameters of the ω-Fe are a**_ω_**_-Fe_ = √2a_α-Fe_, c**_ω_**_-Fe_ = √3/2a_α-Fe_, with α = 90°, β = 90^o^ and γ = 120^o^. Furthermore, the short nano-twinned boundary arranged along the trace of P_1_ (see the purple line in Figure 5h) was identified to be along the ($\bar{2}\bar{1}$1) plane of the α-Fe_marix_ and α-Fe_twin_ (see the two purple vertical lines in Figure 5i). Moreover, the lath boundary arranged along the trace of P_2_ (see the blue line in Figure 5h) was indexed to be along the ($\bar{2}$1$\bar{1}$) plane of α-Fe_matrix_ (see the two blue vertical lines in Figure 5i). In general, the boundaries of the short-twinned bundles and the lath bundles were both confirmed to be suitably generated along the {112} planes of α-Fe.

### 3.2. Morphology and Crystallography of the As-Tempered Martensite

**Figure 6:**
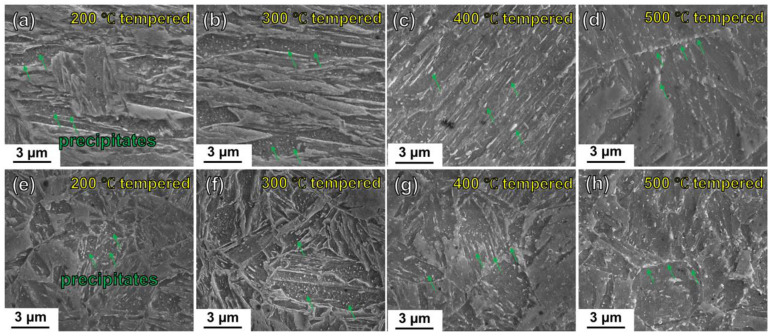
SEM observations of the DQ-treated and CQ-treated specimens after tempering: (**a**–**d**) scanning electron micrographs of the DQ-treated specimens after 200, 300, 400 and 500 °C tempering, respectively; (**e**–**h**) scanning electron micrographs of the CQ-treated specimens after 200, 300, 400 and 500 °C tempering, respectively.

The DQ-induced and CQ-induced lath martensite could be significantly evolved during the tempering at various temperatures. Figure 6a–d show the scanning electron micrographs of the DQ-induced lath martensite after tempering at 200, 300, 400 and 500 °C, respectively. From the figures, the as-tempered martensite exhibited the lath width getting apparently larger with the increase of the tempering temperature. In addition, large amounts of bright-white particles (see the indication of green arrows in Figure 6a) were observed in the laths of the 200 °C-tempered specimen. The bright-white particles became larger in size and fewer in amount in the 300 °C-tempered martensitic laths (see the indication of green arrows in Figure 6b). Additionally, the particles appeared as rod-like shape arranged parallel to the lath boundaries of the 400 °C-tempered martensite (see the indication of green arrows in Figure 6c). In terms of the 500 °C-tempered martensite, the particles have the spherical tendency at the prior austenitic grain boundaries (see the indication of green arrows in Figure 6d). Figure 6e–h show the scanning electron micrographs of the CQ-treated specimens after 200, 300, 400 and 500 °C tempering, respectively. From the figures, the lath morphologies of the tempered martensite tended to disappear with the tempering temperature increasing. In addition, the tempering-temperature-induced evolution of the bright-white particles in the CQ-induced fine-grained martensite during tempering presented a similar tendency to that of the DQ-induced coarse-grained martensite. 

**Figure 7:**
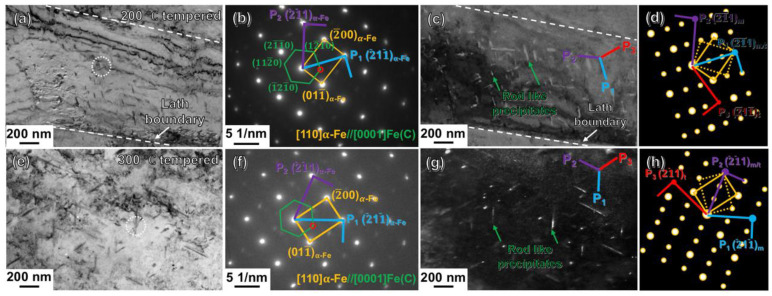
Typical TEM results of the CQ-treated specimens after 200, 300 °C tempering: (**a**,**e**) bright-field images of the 200 °C and 300 °C-tempered martensite, respectively; (**b**,**f**) the corresponding SAED patterns of the precipitated areas inside the tempered martensitic laths of (**a**) and (**e**), respectively; (**c**,**g**) the dark-field images of Fe(C) precipitates corresponding to the areas of (**a**) and (**e**), respectively; (**d**,**h**) the schematic illustrations revealing the {112}<111>-type twinned diffractions referring to the patterns of (**b**) and (**f**), respectively.

In order to further characterize the particles, the TEM observations were carried out on the CQ-treated specimens after tempering treatments. From the TEM observations, no {112}<111>-type nanotwins remained in the as-tempered specimens, indicating that the tempering induced a complete detwinning. Figure 7a shows the bright-field image of the CQ-treated specimen after 200 °C tempering, revealing that the particles precipitated in the as-tempered martensitic laths appeared as rod-like shapes. On the basis of the set of spots with lower brightness in the diffraction pattern of Figure 7b obtained through the SAED at the area circled by the white dotted line in Figure 7a, the rod-like particles were confirmed to be the HCP structured Fe(C) phase. The Fe(C) is the same phase as that precipitated in the DQ-induced lath martensite. Another major set of spots with higher brightness was indexed to be α-Fe in a [110] projection. Figure 7c shows the dark-field image of the Fe(C) precipitates obtained by making one diffraction spot of the Fe(C) phase (see the red circled spot in Figure 7b) pass through the objective aperture. From Figure 7c, the Fe(C) precipitates have an average length value of ~68.38 nm. Additionally, the rod-like Fe(C) precipitates dominantly arranged along three planes of P_1_, P_2_ and P_3_ (see the blue, purple and red line, respectively, in Figure 7c). As schematically illustrated in Figure 7d, the P_1_ and P_2_ arranged along the ($\bar{2}\bar{1}$1) plane (see the two blue vertical lines) and the ($\bar{2}1\bar{1}$) plane (see the two red vertical lines) of the α-Fe_matrix_. Additionally, the P_3_ was exactly along the trace of the ($\bar{2}1\bar{1}$) plane of the imaginary α-Fe_twin_ (see the two purple vertical lines). Figure 7e shows the bright-field image of the CQ-treated specimen after 300 °C tempering treatment. It could be clearly seen that the 300 °C tempering induced the precipitation of the rod-like particles, which were also in the Fe(C) phase, on the basis of the corresponding SAED pattern in Figure 7f. Figure 7g shows the dark-field image of the Fe(C) precipitates obtained using the (1$\bar{2}$10) spot in the [0001] axis of the Fe(C) phase (see the red circled spot in Figure 7f), illustrating that the rod-like Fe(C) tended to become larger in the mean length (~119.53 nm). The rod-like Fe(C) precipitates also dominantly arranged along three planes of P_1_, P_2_ and P_3_ (see the blue, purple and red line in Figure 7g, respectively). The three planes were all along the {112} planes of the α-Fe_matrix_ and the imaginary α-Fe_twin_, as schematically illustrated in Figure 7h. On the basis of the crystallography of the rod-like Fe(C) precipitates, there should be some potential correlations between the generation of the {112}-orientated Fe(C) precipitates and the {112}-type nanotwins. This is going to be discussed later. 

**Figure 8:**
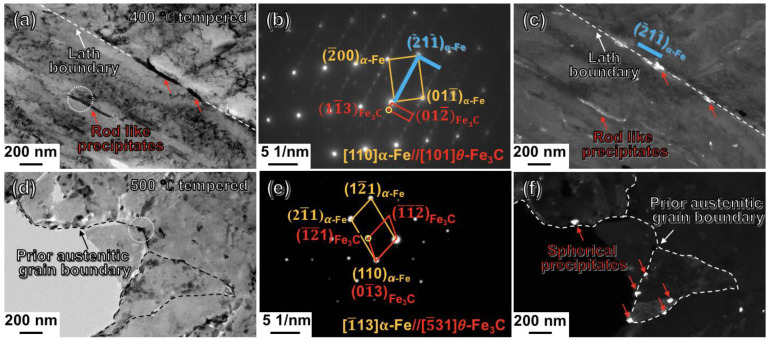
Typical TEM results of the CQ-treated specimens after 400, 500 °C tempering treatments: (**a**,**d**) bright-field images of the 400 °C and 500 °C-tempered martensite, respectively; (**b**,**e**) the corresponding SAED patterns of the precipitated areas inside (**a**) and (**d**), respectively; (**c**,**f**) the dark-field images of Fe_3_C precipitates corresponding to the areas of (**a**) and (**d**), respectively.

The TEM determinations of the CQ-treated specimens after tempering at 400 °C and 500 °C are shown in Figure 8a–c and d–f, respectively. From Figure 8a, the rod-like particles precipitated in the 400 °C-tempered martensitic laths were apparently larger in not only the length, but also the width, in comparison to those in the 300 °C-tempered martensite. Additionally, the particles became apparently smaller in quantity. The SAED pattern of the white-dotted line-circled area in Figure 8a was shown in Figure 8b. The major set of diffraction spots with higher brightness originated from α-Fe along the [110] zone axis. The rod-like particles were indexed to be Fe_3_C (see the red rectangular line connected spots in Figure 8b). The Fe_3_C presented an orientation relationship of [101]Fe3C//[110]α_-Fe_ between the tempered martensite matrix. The dark-field image of the Fe_3_C precipitates obtained through the (1$\bar{1}$3) spot of the Fe_3_C phase (see the yellow circled spot in Figure 8b) is shown in Figure 8c. It can be clearly seen from Figure 8c that the Fe_3_C precipitates, as indicated by the red arrows, generated along the trace of the lath boundary. Curiously, the lath boundary was exactly along the ($\bar{2}$1$\bar{1}$) of the tempered martensitic matrix of α-Fe (see the two blue vertical lines in Figure 8c). Figure 8d shows the bright-field image of the CQ-treated specimen after 500 °C tempering. The result revealed that the lath, block boundaries almost disappeared, and nearly only the prior austenitic grain boundaries outlined by the black dotted line in Figure 8d remained in the 500 °C-tempered martensite. Moreover, the particles appearing as a spherical shape were also confirmed to be Fe_3_C, through indexing the set of spots with lower brightness connected by the red lines in Figure 8e. Figure 8f shows the dark-field image of the Fe_3_C particles obtained through the yellow circled ($\bar{1}\bar{2}$1) spot of Fe_3_C in Figure 8e. The Fe_3_C particles precipitated at the prior austenitic grain boundaries of the 500 °C-tempered martensite are clearly visible to be a bright-white color and spherical-like shape in Figure 8f. It is also apparent that the Fe_3_C particles became shorter in length, in comparison to those in the 400 °C-tempered martensite (Figure 8c). The structural characteristics of the as-quenched and as-tempered lath martensite in the differently heat-treated 12CrNi2 were listed in Table 2.

### 3.3. Mechanical Properties of the Heat-Treated Steels

**Figure 9:**
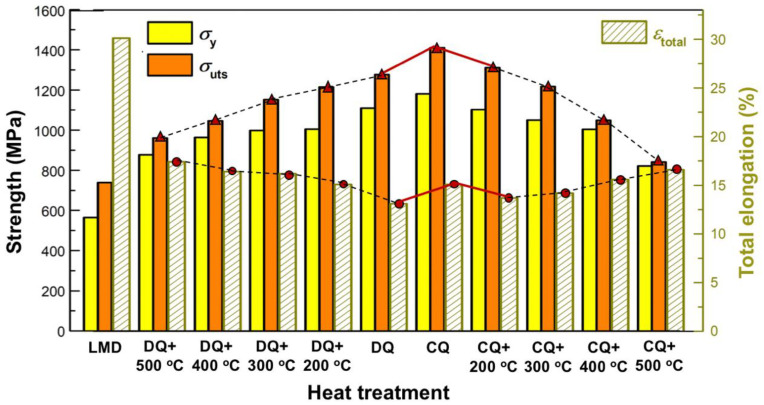
The bar chart illustrates the mechanical properties of the as-deposited specimens before and after the heat treatments including DQ, CQ and the followed tempering.

Figure 9 shows the bar chart illustrating the mechanical properties of the as-deposited and the heat-treated 12CrNi2 alloy steels. The as-deposited specimen had a measured yield strength (σ_y_) of 564.4 MPa, ultimate tensile strength (σ_uts_) of 739.5 MPa, and a total elongation (ε_total_) of 30.1%. The DQ led the as-deposited specimen to have an apparently increased σ_uts_ of 1276.3 MPa, however a lower ε_total_ of 13.1%. It could be clearly seen from Figure 9 that the DQ-treated specimen after tempering at 200 °C to 500 °C for 2 h exhibited a gradually decreased σ_uts_, but enhanced ε_total_. The σ_uts_ of the DQ-treated steel decreased faster when tempering at 400 °C and 500 °C.

In comparison to the DQ-treated steel, the CQ-treated 12CrNi2 exhibited the synchronous enhancement not only in  σ_uts_ (1410.4 MPa), but also in ε_total_ (15.1%). Curiously enough, the 200 °C tempering led both the σ_uts_ and ε_total_ of the CQ-treated steel to decrease into 1310.6 MPa and 13.7%, respectively. Additionally, the  σ_uts_ of the CQ-treated steel after tempering at the increasing temperature from 200 °C to 500 °C gradually decreased from 1310.6 MPa into 841.4 MPa, and the ε_total_ corresponding increased from 13.7% to 16.6%. The mechanical properties of the DED 12CrNi2 without or with the heat treatments are summarized in Table 3. Such was the above mechanical property variation that the CQ induced the overcoming of the strength–plasticity trade-off of the DED steel specimen, as is significantly marked by red solid lines in Figure 9. This could intrinsically be attributed to the CQ-induced preservation of the nanotwins, which is discussed in the sections to follow through involving the quenching–tempering-dependent microstructural evolutions. 

## 4. Discussions

### 4.1. The Evolution of the Martensite during Quenching and Tempering

The microstructural determinations revealed that the lath martensite was apparently refined by the deliberately designed CQ treatment, because the first-step quenching led the as-deposited microstructure to transform into the lath martensite which offered more sites for the nucleation of austenitic grains during the subsequent austenitization at 860 °C, and the 3 min isothermal holding cannot permit the full growth of the austenitic grains. 

**Figure 10:**
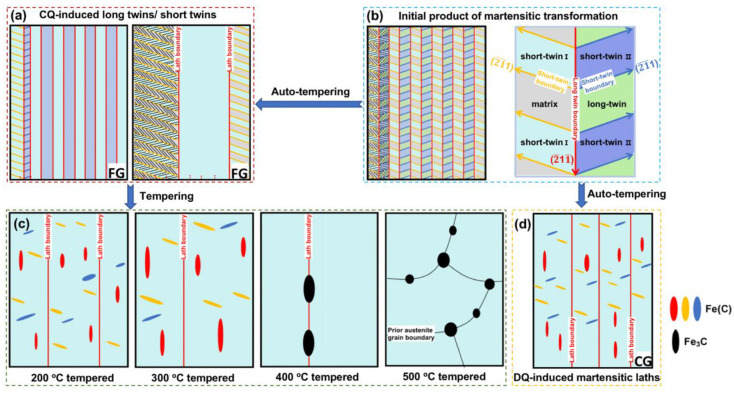
Two-dimensional schematic illustrations revealing the (**a**) CQ-induced long or short twins inside the laths; (**b**) the long and short nested {112}<111>-type twins initially formed during quenching; (**c**) the tempering-temperature-induced evolution of precipitates inside the tempered martensitic laths; (**d**) the DQ-induced rod-like Fe(C) precipitates inside the laths.

The fine-grained lath martensite exhibited a dominant substructure of nanotwins (Figure 5g,h), and the short or long twins are schematically illustrated in Figure 10a. On the basis of the crystallography characteristic (Figure 5i), the long- or short-twinned boundary marked by red or orange lines in Figure 10a were both identified to be along the {112}-twinning planes between the α-Fe_matrix_ and α-Fe_twin_. The α-Fe_twin_ has a definite {112}<111>-type relationship with the α-Fe_matrix_. Moreover, the lath boundary which was inclined to the short-twinned boundary also aligned along the {112} planes of α-Fe (see the two blue vertical lines in Figure 5i). In the other case, the DQ-induced coarse-grained lath martensite exhibited the rod-like Fe(C) particles aligning along three orientations of {112} planes of the α-Fe_matrix_ and α-Fe_twin_. As schematically illustrated in Figure 10d, the red, orange and blue particles represented the rod-like Fe(C) precipitates arranging along different {112} orientations, respectively. Accordingly, it is convincing to believe that the generation of the three {112}-orientated rod-like precipitates could be correlated with the BCC {112}<111>-type nanotwins, which have occurred in the DQ-induced martensite as detected through the TEM (Figure 5d). The correlation could be elaborately deduced as follows. 

The lath martensite was a phase formed when the steel was rapidly water cooling from the single-phase-austenite high temperature, and the martensitic transformation was realized through the lattice transition from FCC γ-Fe to BCC α-Fe, which inevitably caused the volume expansion. From a thermal dynamic point of view, the lattice transition needs strain energy to meet the requirement of the volume expansion as reported by Naghizadeh et al. [28]. In this aspect, it is reasonably assumed that a long- and short-nested twinned structure, as schematically illustrated in Figure 10b, was favorable to be initially generated during the martensitic transformation of DQ and CQ, in order to reduce the strain energy required by the volume expansion of the lattice transition, because the micro-strain generated by the phase-transformation-induced volume expansion was more easily accommodated through the multi-orientation of the twinned structure [29]. Ping et al. also considered that the nested {112}<111>-twinned structure could be the initial product of the martensitic transformation during the water quenching of the low-carbon Fe-C alloys [30]. 

The nano-twins almost disappeared in the DQ-induced lath martensite (Figure 5a,b). This could be intrinsically because the complete detwinning occurred during the auto-tempering of the DQ process. The auto-tempering could be closely associated with the martensitic transformation starting temperature (Ms). A higher Ms always leads to a higher degree of auto-tempering. The Ms value during the DQ (519 °C) was higher than that of the CQ process (395 °C), according to the thermal expansion experimental results. The DQ-induced higher Ms could be related to the larger-sized prior austenitic grains, which always led the lattice transition from γ-Fe to α-Fe to occur more easily at a higher temperature during the martensitic transformation process, because the coarse prior austenitic grain exhibited a lower strength through decreasing the difficulty of the shearing transition [31]. In another aspect, the detwinning occurred incompletely during the auto-tempering of the CQ process, because of the designed CQ-induced finer-grained prior austenitic grains and lower Ms. As a result, longer or shorter nanotwins were retained in the CQ-induced lath martensite (see Figure 5g,h), as schematically shown in Figure 10a. 

Furthermore, the DQ-induced and CQ-induced lath martensite evolved into as-tempered martensite during tempering treatments. Our TEM results confirmed that the long or short nanotwins retained in the CQ-induced martensite completely disappeared, because the tempering treatment naturally led to the complete detwinning. Additionally, the 200°-tempered martensite exhibited rod-like Fe(C) precipitates arranging along the {112} planes of the α-Fe, as schematically illustrated as red, orange and blue colored particles in Figure 10c. The rod-like Fe(C) precipitates may transform from the ω-Fe. The ω-Fe was a phase always distributed at the {112}-twinned boundaries, and had a much larger radius of octahedral interstice (0.43 Å), in comparison to that in the α-Fe lattice (0.19 Å) [32]. As a result, the ω-Fe tended to accommodate more carbon atoms (radius = 0.77 Å) at the {112}-twinned boundaries. Hence, the ω-Fe particles with interstitial carbon atoms transformed into Fe(C) precipitates. And the Fe(C) particles grew into rod-like shape along the original {112}-orientated long- or short-twinned planes. The rod-like Fe(C) precipitates tended to grow to be larger in length along the long axis of the rod during the 300 °C tempering (see Table 2), compared with the 200 °C tempering treatment, because the higher tempering temperature (300 °C) could promote the carbon segregation and the Fe(C) growth along the twinned boundaries [33]. When the tempering temperature increased to 400 °C, rod-like Fe_3_C precipitated from the as-tempered martensite (Figure 8a–c). In simple terms, the Fe_3_C particles, which were transformed from ω-Fe particles in the CQ-induced lath martensite, gathered and developed as rod-like shapes at the lath boundaries during the 400 °C tempering. As the tempering temperature increasing into 500 °C, the laths almost disappeared through the recrystallization of the martensitic laths. The Fe_3_C particles gradually grew, and became coarse and spherical at the effective boundaries of prior austenitic grains of the tempered martensite during the 500 °C tempering. The aforementioned tempering-induced transition is schematically illustrated in Figure 10c.

### 4.2. Effect of Microstructures on Mechanical Properties of the Heat-Treated 12CrNi2

From Figure 9, both the σ_y_ and σ_uts_ of the DQ-treated specimen were significantly higher than those of the as-deposited specimen. The DQ-induced strengthening effect could be intrinsically correlated with the lath martensitic microstructure (Figure 4a,b). Apparently, the lath martensite containing high-density high-angle boundaries of prior austenitic grain, packet and block boundaries (Figure 4c), which were effective barriers hindering the dislocations motion [34]. The DQ-induced martensitic laths also had the substructure of high-density dislocations, which interacted with the stress field produced by the lattice distortion, and hence significantly strengthened the steel through increasing the resistance of the dislocation slipping [35]. Additionally, the Fe(C) particles generated inside the DQ-induced martensitic laths were actually hard precipitates, and they were difficult to cut through by the moving dislocations during the tensile process. The dislocations needed to pass through the precipitates/martensite interfaces through cross slipping [36], which required higher stress compared with the deformation of the major soft ferrite in the as-deposited 12CrNi2 steel. The strengthening mechanisms for the DQ-induced lath martensite were dominated by grain boundary strengthening, dislocation strengthening and precipitation strengthening. However, the strengthening mechanisms made it easier for the dislocations to pile up at the packets, blocks, prior austenitic grain boundaries and the precipitates/martensite interfaces, resulting in a weakened deformation compatibility of the martensite. As a result, the DQ-treated 12CrNi2 exhibited the lowest ε_total_ of 13.1%. 

In contrast to the DQ, the CQ induced the lath martensite to have a higher density of high-angle grain boundaries, because the CQ led the prior austenitic grains, packets and blocks to be significantly refined. The grain refinement naturally caused the enhancement in not only σ_y_ (1180.5 MPa), but also σ_uts_ (1410.4 MPa), according to the Hall–Petch relationship [37]. Additionally, the ω-Fe particles, which were reported to be small-sized in several nanometers and always located at the {112}-twinned boundaries [25], in the CQ-induced martensite apparently increased the stress for the cross-slip movement of the dislocations, and hence provided a developed strengthening effect. Moreover, the nanotwins in the CQ-induced martensite could also hinder the dislocations motion. The nanotwins could also mitigate the dislocations pile-up-induced local stress concentration near the grain boundaries, plausibly because the nano-twinned boundaries could act as effective paths for dislocation slipping, and accommodate more strain there [21,22,38]. As a result of the beneficial effects of the nanotwins on not only strengthening, but also toughening, the CQ-treated 12CrNi2 exhibited a synchronously increased σ_uts_ (1410.4 MPa) and ε_total_ (15.1%). 

From Figure 9, the σ_uts_ and ε_total_ of the CQ-treated steel synchronously decreased into 1310.6 MPa and 13.7% after experiencing a 200 °C tempering treatment. This variation could be discussed through considering the complete detwinning during the 200 °C tempering. Because the nanotwins completely disappeared in the 200 °C-treated steel (Figure 7a), it is easy to understand that the strengthening and toughening effects of the nanotwins in the CQ-treated steel were both eliminated. Additionally, the rod-like Fe(C) precipitates, compared with the ω-Fe particles in the CQ-induced martensite, also provided a weakened strengthening effect, because the Fe(C) precipitates were larger in size and smaller in amount (Figure 7c). In contrast, the rod-like Fe(C) precipitates inside the 300 °C-tempered martensitic laths became longer in length (Table 2), and hence the Fe(C) precipitation-strengthening effect on the steel decreased. Additionally, more dislocations could be released through the higher-temperature (300 °C) tempering. As a result, the σ_uts_ decreased into 1217.4 MPa. 

The σ_uts_ of the CQ-treated 12CrNi2 exhibited a relatively fast decrease after tempering at 400 °C and 500 °C. This could be tied to several factors. First, the precipitation strengthening may be mitigated, because the Fe_3_C precipitates in the 400, 500 °C-tempered martensite, in contrast to the Fe(C) particles in the 200, and the 300 °C-tempered martensite exhibited larger lattice mismatches with the tempered martensitic matrix (α-Fe). The lattice mismatches of Fe_3_C/α-Fe and Fe(C)/α-Fe are both listed in Table 4 [23]. It is apparent that the Fe(C) precipitates had a good fit with the tempered martensitic matrix. The lattice mismatches between the Fe(C) and the α-Fe in the two directions of [001]α-Fe//[1$\bar{2}$10]Fe(C) and [110]α-Fe//[0001]Fe(C) were calculated to be 0.00% by Tirumalasetty et al. On the basis of this calculation, the Fe(C) phase is semi-coherent with the martensitic matrix [23]. The strengthening through the dislocations moving across the stress field produced by the interaction between dislocations and the semi-coherent Fe(C)/α-Fe interfaces could be effectively developed. In contrast, the Fe_3_C particles had a higher lattice misfit with the tempered martensitic matrix. Hence, the Fe_3_C, compared with the Fe(C), could less effectively strengthen the steel, resulting in a sharper reduction of σ_uts_ of the 400 °C-treated specimen. Also, the Fe_3_C particles became spherical and coarse when the tempering temperature increased into 500 °C, and the strengthening could be further weakened. As a result of one of the factors for the toughening mechanisms discussed above, the ε_total_ of the CQ-treated 12CrNi2 after tempering at 400, 500 °C gradually increased (Figure 9). The mechanical properties of the DQ-treated specimens after tempering at various temperatures have a nearly similar variation tendency with the CQ-treated specimen after tempering. Except that the ε_total_ of the DQ-treated steel increased after being tempered at 200 °C, this could be mainly related to the softening of the tempered martensite matrix through the recovery of high-density dislocations, which mitigated the dislocation pile-up at the grain boundaries. 

## 5. Conclusions

A ferrite–austenite 12CrNi2 additively manufactured by DED was, as expected, designed to have fine-grained lath martensite with the main substructure of {112}<111>-type nanotwins and coarse-grained lath martensite with high-density dislocations through the deliberately designed cyclic quenching (CQ) and the direct quenching (DQ). By comparing the tempering-induced evolution of the microstructure and the mechanical property of the CQ-treated steel with that of the direct quenching (DQ)-treated steel, the key conclusions are summarized as follows.

1. The DQ promoted the as-deposited 12CrNi2 to significantly increase in σ_uts_ from 739.5 MPa to 1276.4 MPa, and the decrease of ε_total_ from 30.1% to 13.1%. In contrast to DQ, the CQ led the 12CrNi2 to form a finer-grained lath martensite having numerous {112}<111>-type long or short nanotwins, causing the synchronous increase of σ_uts_ from 1276.4 MPa to 1410.4 MPa, and the ε_total_ from 13.1% to 15.1%.
2. The nanotwins in the CQ-induced lath martensite completely degenerated after tempering at 200 °C. In addition, the 200 °C tempering also induced the precipitation of rod-like Fe(C) particles. The 200 °C tempering induced microstructural evolution, which led the CQ-treated steel to decrease in not only the σ_uts_ (1310.6 MPa), but also the ε_total_ (13.7%). This was an abnormal variation in terms of that induced by the 200 °C tempering carried on the DQ-treated steel, having dislocated lath martensite.
3. The generation of the 200 °C tempering-induced laths and rod-like precipitates was assumed to be intrinsically in connection with the {112}<111>-type long or short nanotwins in the CQ-induced lath martensite, because the tempering-induced laths and rod-like precipitates were all prone to be generated along the {112} planes of the α-Fe crystal, which was exactly fitted with the crystalline orientation of the long or short nanotwins in the CQ-induced martensite.
4. With the tempering temperature increasing into 400, 500 °C, the rod-like Fe(C) transformed into Fe_3_C, and tended to be a spherical shape, resulting in the faster decline of tensile strength. This could be attributed to the fact that the Fe_3_C, in comparison to the Fe(C), exhibited a weaker reinforcement effect, because the lattice mismatch between as-tempered martensitic matrix of α-Fe and Fe_3_C precipitates was relatively higher.

## Figures and Tables

**Table 1:** The average sizes of the hierarchical martensitic microstructures of the DQ-treated and CQ-treated specimens.

Specimens	Prior Austenitic Grain Size (μm)	Packet Size (μm)	Block Width (μm)	Lath Width (nm)
DQ-treated	15.4 ± 1.20	8.25 ± 1.31	2.31 ± 0.56	330.46 ± 41.16
CQ-treated	3.64 ± 0.54	2.33 ± 0.60	0.97 ± 0.36	150.53 ± 17.02

**Table 2:** The summary of the as-quenched and as-tempered martensitic structural characteristics in the heat-treated specimens.

Specimens	Lath Width (nm)	Lath Substructure Type	Precipitates	Precipitate Size (nm)
DQ-treated	330.46 ± 41.16	Dislocations	Fe(C)	38.76 (in length)
CQ-treated	150.53 ± 17.02	Nanotwins	ω-Fe	-
200 °C-tempered	452.01 ± 103.75	Dislocations	Fe(C)	68.38 (in length)
300 °C-tempered	551.83 ± 170.34	Dislocations	Fe(C)	119.53 (in length)
400 °C-tempered	820.06 ± 150.47	Dislocations	Fe_3_C	232.28 (in length)
500 °C-tempered	-	Dislocations	Fe_3_C	37.74 (in diameter)

**Table 3:** Mechanical properties of the as-deposited specimens before and after the heat treatments including the DQ, CQ and the following tempering.

Specimens	*σ*_y_, MPa	*σ*_uts_, MPa	*ε*_total_, %
As-deposited	564.4	739.5	30.1
DQ-treated	1109.6	1276.4	13.1
200 °C-tempered	1004.6	1214.4	15.1
300 °C-tempered	998.4	1151.8	16.2
400 °C-tempered	963.4	1045.7	16.4
500 °C-tempered	877.4	961.3	17.4
CQ-treated	1180.5	1410.4	15.1
200 °C-tempered	1102.5	1310.6	13.7
300 °C-tempered	1049.8	1217.4	14.2
400 °C-tempered	1004.5	1049.3	15.6
500 °C-tempered	821.5	841.4	16.6

**Table 4:** The lattice mismatch between the as-tempered martensite matrix of α-Fe and the precipitates of Fe(C) and Fe_3_C.

Precipitates	Directions	Lattice Mismatch (η $=\left|(d_{\mathrm{precipitates}}-d_{\alpha -Fe})/d_{\alpha -Fe}\right|)$
Fe(C)	[001]α-Fe//[1$\bar{2}$10]Fe(C)	η = 0.00%
	[$\bar{1}$10]α-Fe//[10$\bar{1}$0] Fe(C)	η = 22.3%
	[110]α-Fe//[0001] Fe(C)	η = 0.00%
Fe3C	[0$\bar{1}$1]α-Fe//[100]Fe3C	η = 20.61%
	[211]α-Fe//[001]Fe3C	η = 3%

## Data Availability

The data presented in this study are available on request from the corresponding author.
